# The Effectiveness of Botulinum Toxin Type A (BoNT-A) Treatment in Brazilian Patients with Chronic Post-Stroke Spasticity: Results from the Observational, Multicenter, Prospective BCause Study

**DOI:** 10.3390/toxins12120770

**Published:** 2020-12-04

**Authors:** Patricia Khan, Marcelo Riberto, João Amaury Frances, Regina Chueire, Ana Cristina Ferreira Garcia Amorim, Denise Xerez, Tae Mo Chung, Lucia Helena Costa Mercuri, Alexandre Luiz Longo, Sérgio Lianza, Pascal Maisonobe, Viviane C. Ruiz-Schutz

**Affiliations:** 1 Centro Catarinense de Reabilitação, Florianópolis, Santa Catarina 88025-301, Brazil; patskh@yahoo.com; 2 Faculdade de Medicina de Ribeirão Preto, Universidade de São Paulo, Ribeirão Preto, São Paulo 14049-900, Brazil; mriberto@usp.br; 3 Hospital Bettina Ferro de Souza, Campus IV da Universidade Federal do Pará, Belém, Pará 66075-110, Brazil; amaury.frances@yahoo.com.br; 4 Faculdade de Medicina de São José do Rio Preto, Autarquia Estadual, São José do Rio Preto 15090-000, Brazil; reginahelena.reginahelena@gmail.com; 5 Centro de Reabilitação e Readaptação Dr. Henrique Santillo (CRER), Goiânia, Goiás 74653-230, Brazil; anafisiatra@gmail.com; 6 Serviço de Medicina Física e Reabilitação, Hospital Universitário Clementino Fraga Filho, Universidade Federal do Rio de Janeiro, Rio de Janeiro 21941-590, Brazil; drxerez@gmail.com; 7 Instituto de Medicina Física e Reabilitação, Hospital das Clínicas da Faculdade de Medicina da Universidade de São Paulo, São Paulo 04116-030, Brazil; taemochung@gmail.com; 8 Hospital São Paulo—UNIFESP, São Paulo 04024-002, Brazil; lumercuri1@gmail.com; 9 Clinica Neurológica e Neurocirúrgica de Joinville, Joinville, Santa Catarina 89202-165, Brazil; alexandrellongo@gmail.com; 10 Hospital Alemão Oswaldo Cruz, São Paulo 01323-020, Brazil; sergiol@uol.com.br; 11 Ipsen, 92100 Boulogne Billancourt, France; 12 Ipsen, São Paulo 04571-010, Brazil; viviane.ruiz@ipsen.com

**Keywords:** spasticity, stroke, pain, quality of life

## Abstract

Botulinum toxin type A (BoNT-A) is an effective treatment for post-stroke spasticity; however, some patients cannot access treatment until ≥1 year post-stroke. This Brazilian post-marketing study (NCT02390206) assessed the achievement of person-centered goals in patients with chronic post-stroke spasticity after a BoNT-A injection. Patients had a last documented stroke ≥1 year before study entry and post-stroke upper limb (UL) spasticity, with or without lower limb (LL) spasticity. Patients received BoNT-A injections at baseline (visit 1) and visit 2 (3–6 months). Primary endpoint was responder rate (achievement of primary goal from Goal Attainment Scaling (GAS)) at visit 2. Overall, 204 patients underwent GAS evaluation at visit 2, mean (SD) age was 56.4 (13.2) years and 90.7% had LL spasticity. Median (range) time between first stroke and onset of spasticity was 3.6 (0−349) months, onset of spasticity and first injection was 22.7 (0−350) months and waiting time for a rehabilitation appointment was 9.0 (1−96) months. At visit 2, 61.3% (95% CI: 54.4, 67.7) of patients were responders, which was similar for UL and LL primary goals (57.8% [95% CI: 49.9, 65.3] vs. 64.1% [95% CI: 48.4, 77.3]). This study provides evidence to support the effectiveness of BoNT-A treatment for chronic post-stroke spasticity.

## 1. Introduction

Post-stroke spasticity has been reported to occur in 17–38% of stroke cases [1,2,3]. Botulinum toxin type A (BoNT-A) injections are a known effective treatment for spasticity and are often recommended in clinical practice [4,5,6]. Studies have shown that the use of BoNT-A in the treatment of upper limb (UL) and lower limb (LL) spasticity reduces muscle tone and spasticity symptoms, and improves range of motion (ROM) and functions such as walking speed [7,8,9,10,11]. These physical improvements may enhance the patient’s quality of life (QoL) and reduce the perceived burden on caregivers. BoNT-A products are approved for the treatment of UL and LL spasticity in adults in a number of countries worldwide; in Brazil, the first marketing authorization was granted for onabotulinumtoxinA in 2000 [12]. Treatment with BoNT-A is often deferred until the patient shows clinical signs of spasticity, usually at least 3 months post-stroke [13]. However, recent studies have demonstrated that treatment with BoNT-A as early as 2–12 weeks following stroke may prevent the development of spasticity and improve muscle tone, thus improving prognosis [13,14,15,16].

Although standard practice and treatment guidelines recommend that rehabilitation programs start early in the management of post-stroke spasticity [6], in many low- and middle-income countries this approach is not possible. A lack of rehabilitation in the standard of care, inadequate physician knowledge of the role of rehabilitation, and poor public insurance or financial support, among other contextual factors, are barriers to patients accessing treatment [17]. Consequently, many patients with post-stroke spasticity only receive rehabilitation treatment and BoNT-A in the chronic phase (more than 1 year post-stroke). In clinical practice, there are few studies directly investigating the effectiveness of BoNT-A when first administered during chronic spasticity; therefore, it is not clear whether late administration is effective at reducing disease burden.

The BCause study (Botulinum toxin in the treatment of Chronic post-stroke spAStic patiEnts; NCT02390206) evaluated the achievement of individual person-centered goals, using Goal Attainment Scaling (GAS) [18]. after treatment with BoNT-A injections in Brazilian patients with untreated chronic post-stroke spasticity. The study also aimed to document the time between the onset of spasticity and access to treatment, the sociodemographic profile of the patients, and the standard of care, to identify potential barriers responsible for the delay in receiving adequate treatment.

## 2. Results

### 2.1. Study Population

**Figure 1:**
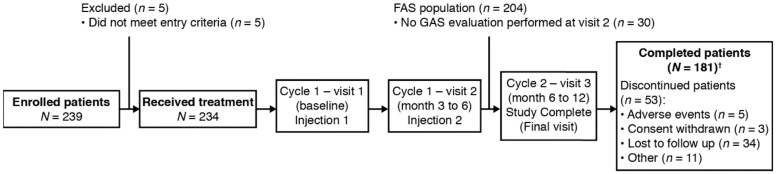
Study design and patient disposition. ^†^ One patient who completed the study and attended visit 3 was not included in the FAS population. FAS, full analysis set; GAS, goal attainment scaling.

A total of 239 patients were enrolled in the study (Figure 1); of the 234 patients who received treatment, 30 did not undergo GAS evaluation at visit 2 (FAS: *n* = 204) and 181 patients completed the study. There were no major protocol deviations/violations in the study.

Participants’ baseline characteristics are reported in Table 1. The mean (standard deviation (SD)) age of patients was 56.4 (13.2) years, 51.5% were male, and 77.9% of patients had experienced only one stroke episode. Patients had a median (range) of 3.6 months (0−349) between their first stroke and onset of spasticity, and over 90% of patients were also affected by spasticity in the LL. The median (range) time between the onset of spasticity and first BoNT-A injection at baseline was 22.7 (0−350) months.

Post-stroke complications were common in patients prior to entry into the study, with most (73.0%; *n* = 149) experiencing at least one complication. Common post-stroke complications included hypertension (53.0%; *n* = 79), depression/anxiety (47.7%; *n* = 71), and aphasia (34.9%; *n* = 52), which were ongoing at baseline in 98.7% (*n* = 78/79), 88.7% (*n* = 63/71) and 59.6% (*n* = 31/52) of patients, respectively.

In total, 25 patients (12.3%) reported using concomitant medications, of whom 19 (76.0%) used baclofen and 171 (83.8%) reported using at least one concomitant non-drug therapy, with 140 patients (77.3%) receiving physical therapy (Figure S1).

### 2.2. Primary Efficacy Endpoint

At visit 2, 125 patients (61.3%; 95% CI: 54.4, 67.7) achieved their primary goal and were considered responders (Table 2), which is above the proportion expected in the sample size calculation. For those patients who set an UL primary goal, 57.8% (95% CI: 49.9, 65.3) were responders compared with 64.1% (95% CI: 48.4, 77.3) who set a LL primary goal.

### 2.3. Secondary Effectiveness Endpoints

#### 2.3.1. Responders at Visit 3

In total, 180 patients (88.2%) attended visit 3, with 56.7% of patients responding to treatment (95% CI: 49.4, 63.8; *n* = 101/178); the proportion of responders was similar in those who set UL and LL primary goals, 57.3% (95% CI: 49.1, 65.2; *n* = 82/143) and 53.1% (95% CI: 36.4, 69.1; *n* = 17/32), respectively. Of the three patients who set a primary goal in both limbs, two were responders (66.7%; 95% CI: 20.2, 94.4; *n* = 2/3).

#### 2.3.2. GAS T Score

**Figure 2:**
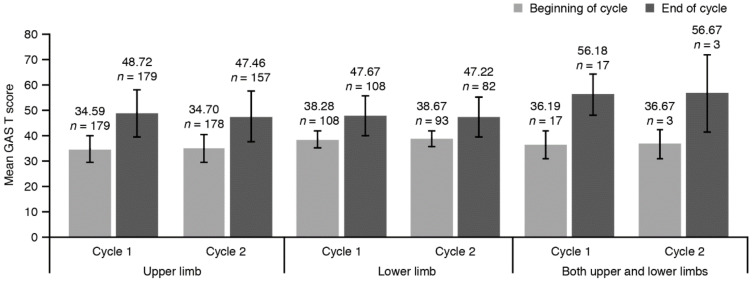
Mean GAS T score during cycles 1 and 2 according to injected limb (FAS). Error bars represent SD. Means are based on the number of non-missing observations in the FAS at the concerned time points. GAS, Goal Attainment Scale; FAS, full analysis set; *n*, number of patients; SD, standard deviation.

GAS T scores increased during cycles 1 and 2 in patients injected in the UL, the LL, and in patients injected in both limbs (Figure 2). The mean (SD) overall cumulated GAS T score across both cycles was 48.24 (8.18; *n* = 191) in patients injected in the UL, 47.17 (6.92; *n* = 123) in patients injected in the LL, and 56.81 (8.13; *n* = 18) in patients injected in both the UL and LL.

#### 2.3.3. Pain Scores

Patients reported their pain scores using a VNS at each visit; at baseline, mean (SD) pain scores were higher at movement than at rest for UL (4.2 [3.8]; *n* = 194 vs. 1.7 [3.1]; *n* = 193, respectively) and LL (2.7 [3.5]; *n* = 173 vs. 1.3 [2.7]; *n* = 172, respectively).

At rest, there was very little change in the mean (SD) pain score in patients injected in the UL from baseline to visit 2 (−0.5 [2.9]; *n* = 193) and from baseline to visit 3 (−0.5 [2.9], *n* = 170). The mean pain score for patients injected in the LL remained stable from baseline to visit 2 (−0.1 [2.7]; *n* = 170) and from baseline to visit 3 (−0.1 [3.0]; *n* = 149).

At movement, the mean (SD) pain score decreased throughout the study in patients injected in the UL, decreasing by −1.4 (3.4) from baseline to visit 2 (*n* = 194) and by −1.7 (3.4) from baseline to visit 3 (*n* = 171). In patients injected in the LL, there was very little change in the mean (SD) pain score from baseline to visit 2 (−0.6 [3.5]; *n* = 170) and baseline to visit 3 (−0.5 [3.5]; *n* = 150).

#### 2.3.4. Range of Motion

Mean (SD) shoulder abduction angle increased throughout the study, increasing by 15.0° (23.0) from baseline to visit 2 (*n* = 150) and by 21.6° (28.6) from baseline to visit 3 (*n* = 129).

#### 2.3.5. MAS Spasticity Scores

The mean MAS scores decreased slightly throughout the study. In the UL, the mean (SD) score decreased by −0.30 (0.50) from baseline to visit 2 and by −0.40 (0.56) from baseline to visit 3, and in the LL decreased by −0.14 (0.54) and by −0.28 (0.59), respectively. Overall, mean (SD) MAS score decreased −0.22 (0.43) from baseline to visit 2 and by −0.34 (0.48) from baseline to visit 3. There was a trend toward a decrease of the MAS score in each UL joint (shoulder, elbow, wrist, finger and thumb) and LL joint (hip, knee, ankle, and toe; Table S1).

#### 2.3.6. Functional Independence

The mean (SD) Barthel Index score increased during cycle 1, from 63.1 (28.5; *n* = 204) to 70.8 (27.3; *n* = 180) with a mean (SD) change of 5.1 (12.3; range: −35 to +50). The proportion of patients who were independent at visit 3 increased compared with baseline for various activities of daily living (Table S2).

**Figure 3:**
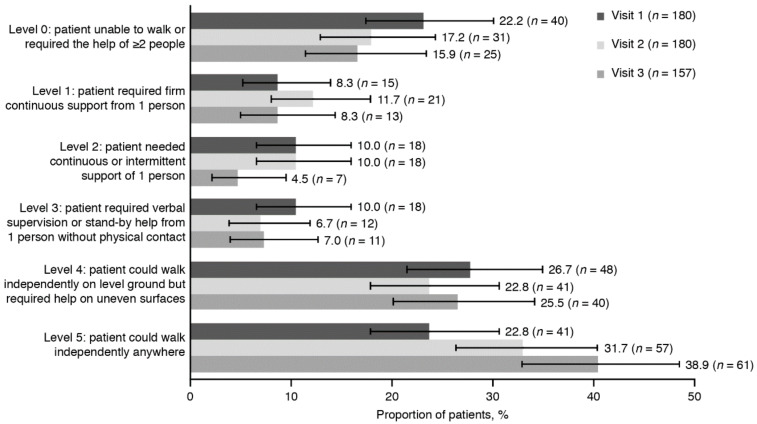
Functional independence according to the FAC classification at visits 1, 2 and 3 (FAS). Error bars represent 95% CI. Percentages are based on the number of non-missing observations in the FAS, data for 24 patients were missing for visits 1 and 2, and data for 47 patients were missing for visit 3. Level 0, non-functional; level 1, dependent level II; level 2, dependent level I; level 3, dependent on supervision; level 4, independent on level ground; level 5, independent. CI, confidence interval; FAC, Functional Ambulation Category; FAS, full analysis set.

Using the FAC classification, the proportion of patients classified as non-functional decreased throughout the study: 22.2% at baseline, 17.2% at visit 2 and 15.9% at visit 3 (Figure 3). Conversely, the proportion of independent patients increased throughout the study: 22.8% at baseline, 31.7% at visit 2 and 38.9% at visit 3.

#### 2.3.7. QoL and Satisfaction

The mean (SD) EQ VAS score increased throughout the study: from 61.8 (24.3; *n* = 137) at baseline to 66.6 (21.6; *n* = 127) at visit 2 and 69.5 (20.8; *n* = 113) at visit 3. The proportion of patients with no problems in walking, washing, or dressing themselves, and doing their usual activities also increased during the study (Table 3), while the proportion of patients with moderate, severe, or extreme pain or discomfort decreased throughout the study.

Investigators, patients, and caregivers also reported on their satisfaction regarding treatment effectiveness, with over 70% of respondents reporting that treatment with BoNT-A provided a great, or some, benefit across cycles 1 and 2 (Table 4).

### 2.4. Socio-Demographic Data

Of the 204 patients included in the FAS, 203 patients provided data on their education history. The majority of patients had attended school (93.1%; *n* = 189), with a median (range) number of years spent attending school of 6.0 (1−18) years, and 24 patients (11.8%) had attended university. The professional status was known for 201 patients: 106 (52.7%) were retired, 71 (35.3%) received social benefits, 11 (5.5%) were unemployed, 8 (4.0%) had never worked, and 5 (2.5%) were employed. The patients’ working status was a consequence of stroke in 63.8% of cases (*n* = 120/188). The majority of patients in the study had public health insurance (90.7%; *n* = 185) compared with private health insurance (9.3%; *n* = 19).

**Figure 4:**
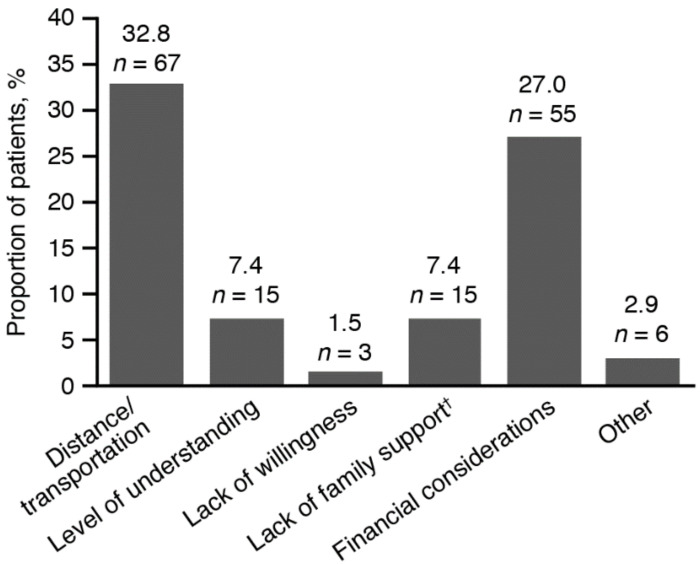
Factors that made receiving treatment difficult for patients (FAS) ^†^ Lack of family support includes having no relative to accompany the patient to appointments. The denominator of percentage is the number of subjects in the FAS. Data for 98 (48.0%) patients in the FAS were missing; the sum of percentages may exceed 100%. FAS, full analysis set.

The most frequent reasons that patients reported for not undergoing earlier rehabilitation were either because rehabilitation was only indicated/prescribed recently (44.6%; *n* = 91), or because they were unable to schedule an appointment for rehabilitation (16.2%; *n* = 33). The median (range) waiting time for an appointment at a center was 9.0 (1−96) months. Patients reported a number of factors that made access to treatment difficult (Figure 4), of which the most common was distance or transportation to the outpatient center (32.8%). The median (range) time for travel to a center was 60.0 (5−240) min.

### 2.5. Injection Practices

Of the 204 patients injected at visit 1 and the 201 patients injected at visit 2, abobotulinumtoxinA was the most frequently injected BoNT-A preparation at both visits (Table 5). The doses of toxin administered to patients according to the injected limbs are shown in Table 5. Overall, the mean (SD) time between injections was 4.6 (0.7) months and using palpation/anatomic landmarks was the most common method used to guide injections. The most frequently injected muscles are reported in Table S3.

### 2.6. Pharmacoeconomic Impact of BoNT-A Injections

In total, 151 (74.0%) patients required a caregiver, frequently a family member (*n* = 129, 85.4%), with only 10 (6.6%) patients having a professional caregiver and 12 (7.9%) a combination of caregivers. Family members and professional caregivers dedicated a mean (SD) of 13.2 (8.6) h (median 12.0 h) and 11.1 (7.3) h (median 10.5 h) per day to the patient, ranging from 1 to 24 h. In total, 49.6% of family members reported that their professional activities were impacted upon by the patient’s disease (*n* = 70/141).

At baseline, the majority of caregivers responded “a little” or “not at all” in regard to physical or psychological burden relating to their patients’ spasticity (Figure S2). The physical burden decreased for 70.2% (*n* = 106/151) of caregivers after cycle 1 and for 63.8% (*n* = 83/130) of caregivers after cycle 2. In comparison, psychological burden decreased for 56.3% (*n* = 85/151) of caregivers after cycle 1 and for 58.5% (*n* = 76/130) of caregivers after cycle 2.

### 2.7. Safety

Overall, 46 SAEs (assessed by sponsor) were reported in 15 patients who had been treated with abobotulinumtoxinA throughout the study; 10 of these SAEs were assessed as possibly related to the study drug, as there was insufficient information for a firm causality assessment (Table S4). Of these 15 patients, there were five deaths, which were all considered as unrelated to the study drug. No new safety findings requiring further investigation were identified during the course of the study.

## 3. Discussion

BCause was an observational, non-interventional, post-marketing study designed to investigate the effectiveness of BoNT-A injections in untreated Brazilian patients with chronic post-stroke spasticity. Here, we provide evidence to support the effectiveness of BoNT-A treatment in this patient population, with over half of patients responding after the first cycle (61.3%) and second cycle (56.7%) of injections indicating the potential benefit of repeat BoNT-A injections in chronic spasticity. The use of injection-guiding techniques, e.g., electrical stimulation or ultrasound, is recommended to target BoNT-A injections [19,20]. In this study, anatomic palpation was used in approximately 90% of patients; this reflects the results from Crema et al. (2016), who reported that 80% of Brazilian medical doctors used muscle palpation techniques and anatomic topographical reference points to guide BoNT-A treatment [21]. Over three-quarters of the patients enrolled in BCause also received physical therapy in addition to BoNT-A injections. In a recent study, Prazeres et al. (2018) reported that BoNT-A injections in combination with physical therapy was superior to physical therapy alone in improving muscle tone in post-stroke spasticity [22].

In comparison to BCause, the ULIS-II study reported that 79.6% of patients with post-stroke UL spasticity achieved, or overachieved, their primary goal from GAS following a single BoNT-A injection cycle [18]. This result is higher than reported in this study, which could be due to differences in the level of goal-setting experience of investigators between the two studies [18]. In ULIS-II, only 40% of patients received their first BoNT-A injection within the first year post-stroke, compared with 60% who received it more than 1 year afterwards [23]. The authors noted that the latter group were older and suggested that younger age could be a trigger for earlier spasticity treatment. Despite this suggestion, the rate of primary goal attainment was similar in both groups (80% vs. 79%, respectively), although a greater proportion of goals relating to active function was achieved by those patients with earlier treatment [23].

In the present study, the effectiveness of BoNT-A was also illustrated by an increase in ROM of the shoulder and decrease in pain in the UL at movement. The use of BoNT-A in the reduction of pain and improvement of ROM in the shoulder has been shown in a number of previous studies [24,25,26], with results from a meta-analysis of randomized controlled trials also reporting that BoNT injections showed more persistent clinical benefits in pain reduction and ROM compared with placebo or steroid injections [27]. The majority (82.8%) of patients in the current study presented with an adducted internally rotated shoulder; Gomes et al. (2019) reported similar results in Brazilian patients with post-stroke motor impairment, with 76% of patients presenting with this pattern of UL spasticity [28]. Improving ROM, motor function, and mobility, and reducing pain are all common goals during rehabilitation post-stroke [29].

The results from the functional independence analyses indicated that patients became more independent throughout the study, with the proportion who could walk independently increasing from 22.8% to 38.9%, and the mean Barthel Index score increasing throughout the study. Pimental et al. (2014) reported that treatment with 100 U or 300 U of BoNT-A improved 10 m walking speed in Brazilian patients with post-stroke LL spasticity [30]. Patients in the current study also documented an improvement in their QoL across all EQ-5D-5L domains at each visit, with the mean EQ VAS score increasing from 61.8 to 69.5 between baseline and visit 3.

The improvement in spasticity symptoms following BoNT-A injections also had a positive impact on caregivers’ physical and psychological burden. This result was also seen by Bhakta and colleagues (2000), who reported a reduction in caregiver burden for at least 12 weeks after a single injection of abobotulinumtoxinA compared with placebo in patients with post-stroke UL spasticity [31]. These outcomes can have both direct and indirect economic impacts on the patient’s families and country. In the current study, approximately 50% of family members found that their professional activities were affected by patients’ spasticity, and over 60% of patients reported that their working status was a consequence of their stroke. Therefore, a meaningful increase in independence could reduce some of this burden. The levels of satisfaction reported by patients and caregivers also support this reduction in disease burden, with approximately 44% of patients and 36% of caregivers reporting a great benefit after two cycles of BoNT-A injections.

Patients reported a number of environmental and personal factors preventing or delaying their access to treatment; the most frequent issues were the distance or transport to a rehabilitation center and being unable to schedule an appointment. These difficulties may be linked to the public healthcare system in Brazil, since over 90% of patients had public health insurance. Jorge and colleagues (2015) noted that there is a shortage of rehabilitation centers in Brazil, along with inadequate knowledge regarding the need for rehabilitation and poor organization between clinical services and rehabilitation centers [32]. A study assessing factors influencing the functional gain in patients post-stroke in Brazil found that the average time from stroke onset until admission to a rehabilitation program was 8.9 months, which was similar to the median waiting time for an appointment at a center in this study (9.0 months) [33]. Carod-Artal and colleagues (2005) noted that, in patients who started a rehabilitation program at a later stage, functional improvement scores were only approximately 73% of those for patients who began treatment during the first 6 months [33].

The main limitation of this study was the high dropout rate of patients; this was due to several factors, including shortages of medication and access to the outpatient centers (which was dependent on both transportation and relatives/caregivers). There were no statistical tests performed on the data from the various assessments; therefore, the dropout rate of patients has not been statistically accounted for. The number of enrolled patients was also slightly lower than the sample size target, which was largely due to the limited amount of time available to enroll patients. Despite this limitation, the proportion of patients who achieved their primary goal was higher than expected. The study was also subject to selection bias as it is likely that those who benefitted most from BoNT-A injections would continue until the end of the study. Furthermore, the majority of patients were treated with abobotulinumtoxinA and, therefore, comparisons between the different BoNT-A formulations could not be made.

## 4. Conclusions

In summary, the results from the BCause study provide evidence that BoNT-A is an effective treatment for patients with untreated chronic post-stroke spasticity in both UL and LL, with over half of patients responding to treatment after a single injection. The results suggest that, in situations where early treatment is not possible, such as in countries with a low socioeconomic status, patients can still benefit from BoNT-A treatment provided they are able to access rehabilitation programs.

## 5. Methods

### 5.1. Study Design

BCause was an observational, multicenter, post-marketing, prospective study conducted at 11 centers in Brazil between June 2015 and August 2017.

### 5.2. Inclusion and Exclusion Criteria

Eligible patients were adults, aged 18–80 years, with a last documented stroke (hemorrhagic or ischemic) at least 1 year prior to study entry. All patients had documented UL spasticity, with or without LL spasticity, and must not have received any prior injections of BoNT-A to treat spasticity symptoms. Patients were not eligible to participate if they had undergone previous surgical procedures for spasticity treatment, had received previous phenol injections, or if they may have been indicated to receive phenol during the study. Patients with contraindications to any BoNT-A preparations were excluded.

### 5.3. Treatment

The decision to prescribe BoNT-A was taken prior to, and independently from, the decision to enroll the patient into the study. BoNT-A was prescribed in accordance with routine clinical practice. As this was an observational study, investigators were free to choose the targeted muscles, BoNT-A preparation, injected doses, injection interval, number of injection points, and volume/dosage per point in accordance with local summary of product characteristics and locally agreed therapeutic guidelines.

Patients were treated across two injection cycles: visit 1 (baseline; cycle 1), visit 2 (3−6 months; cycle 1), and visit 3 (6−12 months, cycle 2). Patients received injections of BoNT-A at visits 1 and 2.

### 5.4. Study Assessments and Endpoints

The primary efficacy endpoint was the responder rate, defined as achievement of the primary goal from the GAS at visit 2. An agreed set of goals (one primary and ≤3 secondary) was established at visit 1 by identifying the main problem areas and which limbs these related to (UL, LL, or both). Each goal was rated as either “some function” (−1) or “no function” (−2). At visit 2, achievement for each goal was recorded using GAS-Light (a verbal rating scale) [34,35], which was converted to a numerical scale (−2, got worse; −1/−2, no change; −0.5, partially achieved; 0, as expected; +1, a little more; +2, a lot more). Patients were considered as responders if they achieved or overachieved their primary goals at visit 2 (scores 0/+1/+2).

Secondary efficacy endpoints assessed the effectiveness of BoNT-A, including the overall attainment of treatment goals using the GAS Total (GAS T) score after each cycle and the overall cumulated GAS T score of all goals assessed during the study. Pain at rest and movement (scored using a visual numeric scale (VNS); from 0 (no pain) to 10 (extreme pain)), ROM using a goniometer at the shoulder level (by measuring the shoulder abduction angle), and Modified Ashworth Scale (MAS) spasticity score (six grades: 0, 1, 1+, 2, 3, or 4 (for quantitative analyses, grade 1+ was considered as 1.5) [9,10,36]) for UL and LL at each joint, were evaluated at all visits. Functional independence was assessed using the Barthel Index score (ordinal scale used to measure performance in 10 variables describing activities of daily living) at visits 1 and 3 and Functional Ambulation Category (FAC) classification, which organized patients into six categories from level 0 (non-functional) to level 5 (independent), at all study visits. Patients’ QoL was also assessed at visits 1, 2 and 3 using the European Quality of Life-5 Dimensions-5 Levels (EQ-5D-5L) questionnaire, where scores were provided using the European Quality of Life Visual Analogue Scale (EQ VAS). Overall satisfaction with treatment effectiveness according to the patient, caregiver, and investigator was assessed at visits 2 and 3 using a 5-point Likert Scale (from “great benefit” to “much worse”).

In addition, a number of secondary endpoints described the patient population and treatment practices; these endpoints included any relevant sociodemographic data at visit 1, and injection practices (e.g., BoNT-A preparation and injection guidance technique). The pharmacoeconomic impact of BoNT-A injections was also assessed at all visits; these included caregiver physical and psychological burden (rated as “not at all”, “a little”, or “a lot”) and the indirect costs of treatment.

This was a non-interventional study in which BoNT-A was administered and managed within routine medical care. Treatment-related adverse events (AEs) and serious AEs (SAEs) were monitored at each study visit and reported to the sponsor’s, or the relevant manufacturer’s, pharmacovigilance department or marketing authorization holder.

### 5.5. Statistical Methods

Sample size was determined based on the primary efficacy endpoint. Based on a literature review, it was expected that at least 60% of patients treated with BoNT-A would respond after one cycle of treatment (21). A minimum of 219 patients was needed to achieve a fixed precision of 6.5% for a 2-sided 95% confidence interval (CI) for the expected response rate of 60%, using normal approximation. Assuming a 10% expected dropout rate, a total number of 244 patients was required.

The full analysis set (FAS) included all patients who received at least one injection of BoNT-A and underwent at least one post-baseline assessment of the primary efficacy parameter. Both primary and secondary endpoints were conducted using the FAS.

The primary endpoint was summarized by the number and percentage of responders overall and per primary goal limbs, together with 2-sided 95% Agresti–Coull approximate CIs for binomial proportions. For secondary effectiveness endpoints, quantitative summary statistics and/or frequency counts were presented. No statistical analyses of safety data or imputations for missing data were performed.

### 5.6. Ethical Approval

This study was conducted in compliance with the Declaration of Helsinki, the International Ethical Guidelines for Epidemiological Studies, and all local regulatory requirements applicable to non-interventional studies. The protocol was approved by the Ethics Committee of FAMERP—Instituto de Reabilitação Lucy Montoro (Project identification code: A-38-52120-202) on 10 July 2014. Written informed consent (signed by the participant or his/her legal representative) was provided prior to any study-related procedure.

## Figures and Tables

**Table 1:** Baseline characteristics in patients with chronic post-stroke spasticity (FAS). ^†^ The denominator of percentage is the number of patients with affected limbs. Percentages are based on the number of non-missing observations in the FAS. CVA, cerebrovascular accident; FAS, full analysis set; LL, lower limb; SD, standard deviation; UL, upper limb.

Parameter	All Patients (*n* = 204)
Age in years, mean (SD); median [range]	56.4 (13.2); 59.0 [18−79]
Sex, *n* (%) Male Female	105 (51.5) 99 (48.5)
Handedness, *n* (%) Ambidextrous Left Right	4 (2.0) 14 (6.9) 186 (91.2)
Time since last CVA (months), mean (SD); median [range]	51.3 (61.4); 28.1 [12−374]
Etiology of last CVA, *n* (%) Hemorrhage Infarct Infarct and hemorrhage	45 (22.1) 151 (74.0) 8 (3.9)
Location of last CVA, *n* (%) Left hemisphere Posterior circulation Right hemisphere	99 (48.5) 3 (1.5) 102 (50.0)
Number of CVA episodes (previous and studied), *n* (%) 1 2 3 >3	159 (77.9) 32 (15.7) 8 (3.9) 5 (2.5)
Time since onset of stroke (months), mean (SD); median [range]	61.6 (69.4); 32.0 [12−374]
Time since onset of spasticity (months), mean (SD); median [range]	40.2 (53.1); 22.7 [0−350]
Time between first CVA and onset of spasticity (months), mean (SD); median [range]	25.7 (53.2); 3.6 [0−349]
UL affected by spasticity, *n* (%) Right Left Bilateral	204 (100) 93 (45.6) 105 (51.5) 6 (2.9)
LL affected by spasticity, *n* (%) Right Left Bilateral	185 (90.7) 84 (45.4) 95 (51.4) 6 (3.2)
UL spasticity patterns, *n* (%) ^†^ Adducted internally rotated shoulder Flexed elbow Flexed wrist Pronated forearm Clenched fist Thumb-in-palm	169 (82.8) 168 (82.4) 144 (70.6) 133 (65.2) 113 (55.4) 101 (49.5)
LL spasticity patterns, *n* (%) ^†^ Equinovarus foot Knee extension Claw toes Knee flexion Hip adduction Hip flexion Striatal toe	147 (79.5) 89 (48.1) 70 (37.8) 52 (28.1) 36 (19.5) 31 (16.8) 23 (12.4)

**Table 2:** Primary efficacy endpoint (FAS). A responder was defined as a patient who achieved or overachieved the primary goal (goal rating of 0, +1, or +2). The response after the first injection cycle was assessed at visit 2. Percentages are based on the number of patients with primary goal as applicable for the different limbs. FAS, full analysis set; LL, lower limb; n, number of patients; UL, upper limb.

Visit 2	*n* (%)	95% CI
Primary goal limb		
UL (*n* = 154)	89 (57.8)	49.9, 65.3
LL (*n* = 39)	25 (64.1)	48.4, 77.3
Both limbs (*n* = 11)	11 (100.0)	70.0, 100.0
Overall (*n* = 204)	125 (61.3)	54.4, 67.7

**Table 3:** Frequency of reported problems in the five domains of the EQ-5D-5L (FAS). Percentages are based on the number of non-missing observations in the FAS. CI, confidence interval; EQ-5D-5L, European Quality of Life-5 Dimensions-5 Levels; FAS, full analysis set; *n*, number of patients.

Domain	Level	Visit 1, *n* (%) [95% CI]	Visit 2, *n* (%) [95% CI]	Visit 3, *n* (%) [95% CI]
Mobility	I have no problem in walking about	7 (5.1) [2.3, 10.4]	13 (10.2) [6.0, 16.9]	17 (15.0) [9.5, 22.9]
	I have slight problems in walking about	35 (25.5) [19.0, 33.5]	39 (30.7) [23.3, 39.2]	40 (35.4) [27.2, 44.6]
	I have moderate problems in walking about	38 (27.7) [20.9, 35.8]	38 (29.9) [22.6, 38.4]	32 (28.3) [20.8, 37.3]
	I have severe problems in walking about	36 (26.3) [19.6, 34.2]	19 (15.0) [9.7, 22.3]	9 (8.0) [4.1, 14.6]
	I am unable to walk about	21 (15.3) [10.2, 22.4]	18 (14.2) [9.1, 21.4]	15 (13.3) [8.1, 20.9]
	Missing	67	77	91
Self-care	I have no problem washing or dressing myself	25 (18.2) [12.6, 25.6]	34 (26.8) [19.8, 35.1]	45 (39.8) [31.3, 49.0]
	I have slight problems washing or dressing myself	31 (22.6) [16.4, 30.4]	32 (25.2) [18.4, 33.4]	23 (20.4) [13.9, 28.8]
	I have moderate problems washing or dressing myself	29 (21.2) [15.1, 28.8]	30 (23.6) [17.0, 31.8]	25 (22.1) [15.4, 30.7]
	I have severe problems washing or dressing myself	17 (12.4) [7.8, 19.1]	11 (8.7) [4.8, 15.0]	10 (8.8) [4.7, 15.7]
	I am unable to wash or dress myself	35 (25.5) [19.0, 33.5]	20 (15.7) [10.4, 23.2]	10 (8.8) [4.7, 15.7]
	Missing	67	77	91
Usual activity	I have no problems doing my usual activities	9 (6.6) [3.4, 12.3]	15 (11.8) [7.2, 18.7]	21 (18.6) [12.4, 26.8]
	I have slight problems doing my usual activities	22 (16.2) [10.9, 23.3]	36 (28.3) [21.2, 36.8]	34 (30.1) [22.4, 39.1]
	I have moderate problems doing my usual activities	38 (27.9) [21.1, 36.0]	34 (26.8) [19.8, 35.1]	23 (20.4) [13.9, 28.8]
	I have severe problems doing my usual activities	28 (20.6) [14.6, 28.2]	16 (12.6) [7.8, 19.6]	17 (15.0) [9.5, 22.9]
	I am unable to do my usual activities	39 (28.7) [21.7, 36.8]	26 (20.5) [14.3, 28.4]	18 (15.9) [10.2, 23.9]
	Missing	68	77	91
Pain/discomfort	I have no pain or discomfort	40 (29.2) [22.2, 37.2]	47 (37.0) [29.1, 45.7]	53 (46.9) [38.0, 56.1]
	I have slight pain or discomfort	35 (25.5) [19.0, 33.5]	41 (32.3) [24.8, 40.8]	32 (28.3) [20.8, 37.3]
	I have moderate pain or discomfort	34 (24.8) [18.3, 32.7]	24 (18.9) [13.0, 26.6]	18 (15.9) [10.2, 23.9]
	I have severe pain or discomfort	20 (14.6) [9.6, 21.6]	13 (10.2) [6.0, 16.9]	9 (8.0) [4.1, 14.6]
	I am extreme pain or discomfort	8 (5.8) [2.8, 11.3]	2 (1.6) [0.1, 5.9]	1 (0.9) [0.0, 5.3]
	Missing	67	77	91
Anxiety/depression	I am not anxious or depressed	47 (34.3) [26.9, 42.6]	39 (30.7) [23.3, 39.2]	43 (38.1) [29.6, 47.3]
	I am slightly anxious or depressed	36 (26.3) [19.6, 34.2]	38 (29.9) [22.6, 38.4]	36 (31.9) [24.0, 40.9]
	I am moderately anxious or depressed	30 (21.9) [15.8, 29.6]	32 (25.2) [18.4, 33.4]	21 (18.6) [12.4, 26.8]
	I am severely anxious or depressed	10 (7.3) [3.9, 13.1]	12 (9.4) [5.4, 15.9]	10 (8.8) [4.7, 15.7]
	I am extremely anxious or depressed	14 (10.2) [6.1, 16.5]	6 (4.7) [2.0, 10.1]	3 (2.7) [0.6, 7.9]
	Missing	67	77	91

**Table 4:** Satisfaction regarding treatment effectiveness according to investigators, patients, and caregivers (FAS). Percentages are based on the number of non-missing observations in the FAS. CI, confidence interval; FAS, full analysis set; *n*, number of patients.

Visit	Treatment Satisfaction	Investigators’ Global Assessment of Benefit, *n* (%) [95% CI]	Patients’ Global Assessment of Benefit, *n* (%) [95% CI]	Caregivers’ Global Assessment of Benefit, *n* (%) [95% CI]
End of cycle 1	Great benefit	61 (29.9) [24.0, 36.5]	81 (39.7) [33.2, 46.6]	72 (35.3) [29.1, 42.1]
	Some benefit	135 (66.2) [59.4, 72.3]	92 (45.1) [38.4, 52.0]	80 (39.2) [32.8, 46.1]
	Same	7 (3.4) [1.5, 7.0]	22 (10.8) [7.2, 15.8]	16 (7.8) [4.8, 12.4]
	Worse	0 (0) [0.0, 2.2]	2 (1.0) [0.0, 3.7]	0 (0) [0.0, 2.2]
	Much worse	1 (0.5) [0.0, 3.0]	0 (0) [0.0, 2.2]	0 (0) [0.0, 2.2]
	Not done	0 (0) [0.0, 2.2]	7 (3.4) [1.5, 7.0]	36 (17.6) [13.0, 23.5]
	Missing	0	0	0
End of cycle 2	Great benefit	67 (37.2) [30.5, 44.5]	79 (43.9) [36.8, 51.2]	65 (36.1) [29.4, 43.4]
	Some benefit	102 (56.7) [49.4, 63.7]	72 (40.0) [33.1, 47.3]	64 (35.6) [28.9, 42.8]
	Same	7 (3.9) [1.7, 8.0]	19 (10.6) [6.8, 16.0]	12 (6.7) [3.7, 11.4]
	Worse	4 (2.2) [0.7, 5.8]	2 (1.1) [0.0, 4.2]	1 (0.6) [0.0, 3.4]
	Much worse	0 (0) [0.0, 2.5]	1 (0.6) [0.0, 3.4]	0 (0) [0.0, 2.5]
	Not done	0 (0) [0.0, 2.5]	7 (3.9) [1.7, 8.0]	38 (21.1) [15.8, 27.7]
	Missing	24	24	24

**Table 5:** Drug administration at visits 1 and 2 (FAS) BoNT-A, botulinum toxin type A; FAS, full analysis set; LL, lower limb; *n*, number of patients; SD, standard deviation, UL, upper limb.

Injection Parameters	AbobotulinumtoxinA	OnabotulinumtoxinA	Chinese Type A Botulinum Toxin	Other Unspecified BoNT-A Preparation	Overall
Number of patients injected at visit 1, *n* (%)	173 (84.8)	6 (2.9)	3 (1.5)	22 (10.8)	204 (100)
	Dose administered at visit 1, median (range) units				
UL	600.0 (100.0−1500.0)	300.0 (190.0−315.0)	205.0 (205.0−360.0)	197.5 (37.0−355.0)	-
LL	450.0 (80.0−950.0)	300.0 (100.0−350.0)	220.0 (200.0−260.0)	56.0 (20.0−400.0)	-
Both limbs	1000.0 (400.0−1904.0)	600.0 (300.0−600.0)	425.0 (405.0−620.0)	207.5 (99.0−730.0)	-
Number of muscles injected at visit 1, mean (SD)	7.7 (3.2)	9.3 (1.8)	15.0 (1.0)	9.9 (2.4)	8.1 (3.2)
	Injection guidance technique used at visit 1, *n* (%)				
Palpation/anatomic landmarks	156 (90.2)	2 (33.3)	3 (100)	22 (100)	183 (89.7)
Electric stimulation	19 (11.0)	4 (66.7)	0	0	23 (11.3)
Echography/ultrasound	0	0	0	1 (4.5)	1 (0.5)
Number of patients injected at visit 2, *n* (%)	170 (84.6)	6 (3.0)	3 (1.5)	22 (10.9)	201 (100)
	Dose administered at visit 2, median (range) units				
UL	600.0 (50.0−1500.0)	307.5 (185.0−400.0)	245.0 (201.0−460.0)	149.5 (37.0−265.0)	-
LL	500.0 (25.0−1100.0)	237.5 (100.0−315.0)	250.0 (190.0−290.0)	40.0 (20.0−100.0)	-
Both limbs	1000.0 (175.0−1960.0)	550.0 (380.0−600.0)	495.0 (491.0−650.0)	180.0 (99.0−275.0)	-
Number of muscles injected at visit 2, mean (SD)	7.4 (3.4)	9.8 (0.8)	14.7 (1.5)	8.0 (3.0)	7.7 (3.4)
	Injection guidance technique used at visit 2, *n* (%)				
Palpation/anatomic landmarks	154 (90.6)	3 (50.0)	3 (100)	22 (100)	182 (90.5)
Electric stimulation	18 (10.6)	4 (66.7)	0	0	22 (10.9)

## Supplementary Materials

The following are available online at https://www.mdpi.com/2072-6651/12/12/770/s1, Table S1: (A) MAS in UL joints; (B) MAS in LL joints (FAS), Table S2: Barthel Index score for activities of daily living (FAS), Table S3: Most frequently injected muscles at visits 1 and 2 (FAS), Table S4: Summary of serious adverse events and causality in patients receiving abobotulinumtoxinA, Figure S1: Previous and concomitant therapies: (A) concomitant medications for spasticity (*n* = 25); (B) concomitant non-drug therapies for spasticity (*n* = 181 ^†^), Figure S2: Caregiver’s physical and psychological burden at baseline (FAS).
