# Supplementary material for: The Effectiveness of Botulinum Toxin Type A (BoNT-A) Treatment in Brazilian Patients with Chronic Post-Stroke Spasticity: Results from the Observational, Multicenter, Prospective BCause Study
Source: Toxins (Basel). 2020 Dec 4;12(12):770. doi: 10.3390/toxins12120770 (PMC7762077; doi:10.3390/toxins12120770)
Supplement: Supplementary file 1 [file toxins-12-00770-s001.pdf]

Supplementary Materials: The Effectiveness of Botulinum Toxin Type A (BoNT-A) Treatment in Brazilian Patients with Chronic Post-stroke Spasticity: Results from the Observational, Multicenter, Prospective BCause Study

Patricia Khan, Marcelo Riberto, João Amaury Frances, Regina Chueire and Ana Cristina Ferreira Garcia Amorim, Denise Xerez, Tae Mo Chung, Lucia Helena Costa Mercuri, Alexandre Luiz Longo, Sérgio Lianza, Pascal Maisonobe and Viviane C. Ruiz-Schutz

Table S1. Most frequently injected muscles at visits 1 and 2 (FAS).

**Muscle, *n* (%)**	**Visit 1 (*n* = 204)**	**Visit 2 (*n* = 201)**
Pectoralis major	131 (64.2)	114 (56.7)
Flexor digitorum superficialis	133 (65.2)	115 (57.2)
Biceps brachii	119 (58.3)	104 (51.7)
Gastrocnemius—medial and lateral	120 (58.8)	117 (58.2)
Brachioradialis	90 (44.1)	90 (44.8)

The most frequently injected muscles were identified on study level, not on injection-cycle level. FAS, full analysis set.

Table S2. (**A**) MAS in UL joints; (**B**) MAS in LL joints (FAS).

A)

		Shoulder		Elbow		Wrist		Finger		Thumb	
**Visit**	**Score**	***n* (%)**	**95% CI**	***n* (%)**	**95% CI**	***n* (%)**	**95% CI**	***n* (%)**	**95% CI**	***n* (%)**	**95% CI**
Baseline (visit 1)	0	22 (10.9)	7.2, 16.0	6 (3.0)	1.2, 6.4	19 (9.4)	6.0, 14.3	15 (7.4)	4.5, 12.0	41 (20.4)	15.4, 26.5
	1	33 (16.3)	11.8, 22.1	23 (11.3)	7.6, 16.5	33 (16.3)	11.8, 22.1	26 (12.9)	8.9, 18.2	31 (15.4)	11.0, 21.1
	1+	49 (24.3)	18.8, 30.6	42 (20.7)	15.7, 26.8	41 (20.3)	15.3, 26.4	28 (13.9)	9.7, 19.4	42 (20.9)	15.8, 27.1
	2	47 (23.3)	18.0, 29.6	73 (36.0)	29.7, 42.8	52 (25.7)	20.2, 32.2	51 (25.2)	19.7, 31.7	44 (21.9)	16.7, 28.1
	3	50 (24.8)	19.3, 31.2	56 (27.6)	21.9, 34.1	47 (23.3)	18.0, 29.6	71 (35.1)	28.9, 42.0	38 (18.9)	14.1, 24.9
	4	1 (0.5)	0.0, 3.0	3 (1.5)	0.3, 4.5	10 (5.0)	2.6, 9.0	11 (5.4)	3.0, 9.6	5 (2.5)	0.9, 5.9
	Missing	2		1		2		2		3	
Visit 2	0	30 (14.7)	10.5, 20.3	9 (4.4)	2.2, 8.3	29 (14.2)	10.0, 19.7	18 (8.8)	5.6, 13.6	46 (22.5)	17.3, 28.8
	1	49 (24.0)	18.7, 30.3	37 (18.1)	13.4, 24.0	39 (19.1)	14.3, 25.1	43 (21.1)	16.0, 27.2	50 (24.5)	19.1, 30.9
	1+	46 (22.5)	17.3, 28.8	75 (36.8)	30.4, 43.6	53 (26.0)	20.4, 32.4	38 (18.6)	13.9, 24.6	39 (19.1)	14.3, 25.1
	2	53 (26.0)	20.4, 32.4	54 (26.5)	20.9, 32.9	54 (26.5)	20.9, 32.9	58 (28.4)	22.7, 35.0	43 (21.1)	16.0, 27.2
	3	26 (12.7)	8.8, 18.1	26 (12.7)	8.8, 18.1	28 (13.7)	9.6, 19.2	41 (20.1)	15.1, 26.2	21 (10.3)	6.8, 15.3
	4	0	0.0, 2.2	3 (1.5)	0.3, 4.4	1 (0.5)	0.0, 3.0	6 (2.9)	1.2, 6.4	5 (2.5)	0.9, 5.8
	Missing	0		0		0		0		0	
Visit 3	0	27 (15.0)	10.5, 21.0	19 (10.6)	6.8, 16.0	27 (15.0)	10.5, 21.0	19 (10.6)	6.8, 16.0	37 (20.6)	15.3, 27.1
	1	59 (32.8)	26.3, 39.9	41 (22.8)	17.2 29.5	42 (23.3)	17.7, 30.1	34 (18.9)	13.8, 25.3	46 (25.6)	19.7, 32.4
	1+	43 (23.9)	18.2, 30.6	56 (31.1)	24.8, 38.2	50 (27.8)	21.7, 34.7	45 (25.0)	19.2, 31.8	43 (23.9)	18.2, 30.6
	2	34 (18.9)	13.8, 25.3	44 (24.4)	18.7, 31.2	41 (22.8)	17.2, 29.5	50 (27.8)	21.7, 34.7	37 (20.6)	15.3, 27.1
	3	17 (9.4)	5.9, 14.7	20 (11.1)	7.2, 16.6	20 (11.1)	7.2, 16.6	27 (15.0)	10.5, 21.0	12 (6.7)	3.7, 11.4
	4	0	0.0, 2.5	0	0.0, 2.5	0	0.0, 2.5	5 (2.8)	1.0, 6.5	5 (2.8)	1.0, 6.5
	Missing	24		24		24		24		24	

B)

		Hip		Knee		Ankle		Toe	
**Visit**	**Score**	***n* (%)**	**95% CI**	***n* (%)**	**95% CI**	***n* (%)**	**95% CI**	***n* (%)**	**95% CI**
Baseline (Visit 1)	0	81 (45.5)	38.4, 52.8	37 (20.8)	15.4, 27.4	12 (6.7)	3.7, 11.4	74 (41.8)	34.8, 49.2
	1	34 (19.1)	14.0, 25.5	37 (20.8)	15.4, 27.4	16 (8.9)	5.5, 14.0	37 (20.9)	15.5, 27.5
	1+	25 (14.0)	9.6, 20.0	30 (16.9)	12.0, 23.1	25 (13.9)	9.5, 19.8	18 (10.2)	6.5, 15.6
	2	21 (11.8)	7.8, 17.4	46 (25.8)	19.9, 32.8	55 (30.6)	24.3, 37.6	27 (15.3)	10.7, 21.3
	3	15 (8.4)	5.1, 13.5	24 (13.5)	9.2, 19.3	61 (33.9)	27.4, 41.1	17 (9.6)	6.0, 14.9
	4	2 (1.1)	0.0, 4.3	4 (2.2)	0.7, 5.8	11 (6.1)	3.3, 10.7	4 (2.3)	0.7, 5.9
	Missing	26		26		24		27	
Visit 2	0	82 (45.8)	38.7, 53.1	42 (23.0)	17.4, 29.6	8 (4.4)	2.1, 8.5	83 (46.1)	39.0, 53.4
	1	34 (19.0)	13.9, 25.4	36 (19.7)	14.5, 26.1	19 (10.4)	6.7, 15.7	31 (17.2)	12.4, 23.4
	1+	22 (12.3)	8.2, 18.0	45 (24.6)	18.9, 31.3	43 (23.5)	17.9, 30.2	22 (12.2)	8.1, 17.9
	2	31 (17.3)	12.4, 23.6	45 (24.6)	18.9, 31.3	66 (36.1)	29.5, 43.2	27 (15.0)	10.5, 21.0
	3	8 (4.5)	2.1, 8.7	13 (7.1)	4.1, 11.9	42 (23.0)	17.4, 29.6	15 (8.3)	5.0, 13.4
	4	2 (1.1)	0.0, 4.2	2 (1.1)	0.0, 4.2	5 (2.7)	1.0, 6.4	2 (1.1)	0.0, 4.2
	Missing	25		21		21		24	
Visit 3	0	86 (53.1)	45.4, 60.6	48 (29.6)	23.1, 37.1	8 (5.0)	2.4, 9.6	78 (48.2)	40.9, 56.1
	1	33 (20.4)	14.9, 27.3	38 (23.5)	17.6, 30.6	30 (18.6)	13.3, 25.4	29 (18.0)	12.8, 24.7
	1+	19 (11.7)	7.6, 17.7	33 (20.4)	14.9, 27.3	46 (28.6)	22.1, 36.0	19 (11.8)	7.6, 17.8
	2	16 (9.9)	6.1, 15.5	34 (21.0)	15.4, 27.9	48 (29.8)	23.3, 37.3	23 (14.3)	9.6, 20.6
	3	7 (4.3)	1.9, 8.8	8 (4.9)	2.4, 9.6	23 (14.3)	9.6, 20.6	10 (6.2)	3.3, 11.2
	4	1 (0.6)	0.0, 3.8	1 (0.6)	0.0, 3.8	6 (3.7)	1.5, 8.1	2 (1.2)	0.1, 4.7
	Missing	42		42		43		43	

Percentages are based on the number of non-missing observations in the FAS. Scores: 0 = no increase in muscle tone, 1 = slight increase in muscle tone, 1+ = slight increase in muscle tone, 2 = more marked increase in muscle tone, 3 = considerable increase in muscle tone, 4 = affected part is rigid in flexion or extension. FAS, full analysis set; LL, lower limb; MAS, Modified Ashworth Scale; UL, upper limb.

Table S3. Barthel Index score for activities of daily living (FAS).

Item	Visit	Level of Performance	*n* (%)	95% CI
Feeding	Baseline (visit 1)	Unable	11 (5.4)	2.9, 9.5
		Needs help cutting, spreading butter, etc., or requires modified diet	140 (68.6)	62.0, 74.6
		Independent	52 (26.0)	20.4, 32.4
		Missing	0	
	Visit 3	Unable	13 (7.2)	4.2, 12.2
		Needs help cutting, spreading butter, etc., or requires modified diet	93 (51.7)	44.4, 58.9
		Independent	74 (41.1)	34.2, 48.4
		Missing	24	
Bathing	Baseline (visit 1)	Dependent	98 (48.0)	41.3, 54.9
		Independent (or in shower)	106 (52.0)	45.1, 58.7
		Missing	0	
	Visit 3	Dependent	71 (39.4)	32.6, 46.7
		Independent (or in shower)	109 (60.6)	53.3, 67.4
		Missing	24	
Grooming	Baseline (visit 1)	Needs to help with personal care	86 (42.2)	35.6, 49.0
		Independent face/hair/teeth/shaving (implements provided)	118 (57.8)	51.0, 64.4
		Missing	0	
	Visit 3	Needs to help with personal care	58 (32.2)	25.8, 39.4
		Independent face/hair/teeth/shaving (implements provided)	122 (67.8)	60.6, 74.2
		Missing	24	
Dressing	Baseline (visit 1)	Dependent	52 (25.5)	20.0, 31.9
		Needs help but can do about half unaided	101 (49.5)	42.7, 56.3
		Independent (including buttons, zips, laces, etc.)	51 (25.0)	19.5, 31.4
		Missing	0	
	Visit 3	Dependent	32 (17.8)	12.8, 24.1
		Needs help but can do about half unaided	85 (47.2)	40.1, 54.5
		Independent (including buttons, zips, laces, etc.)	63 (35.0)	28.4, 42.2
		Missing	24	
Bowels	Baseline (visit 1)	Incontinent (or needs to be given enemas)	14 (6.9)	4.0, 11.3
		Occasional accident	26 (12.7)	8.8, 18.1
		Continent	164 (80.4)	74.4, 85.3
		Missing	0	
	Visit 3	Incontinent (or needs to be given enemas)	6 (3.3)	1.4, 7.2
		Occasional accident	14 (7.8)	4.6, 12.7

		Continent	160 (88.9)	83.4, 92.8
		Missing	24	
Bladder	Baseline (visit 1)	Incontinent, or catheterized and unable to manage alone	23 (11.3)	7.6, 16.4
		Occasional accident	50 (24.5)	19.1, 30.9
		Continent	131 (64.2)	57.4, 70.5
		Missing	0	
	Visit 3	Incontinent, or catheterized and unable to manage alone	10 (5.6)	2.9, 10.0
		Occasional accident	26 (14.4)	10.0, 20.4
		Continent	144 (80.0)	73.5, 85.2
		Missing	24	
Toilet use	Baseline (visit 1)	Dependent	49 (24.0)	18.7, 30.3
		Needs some help, but can do something alone	46 (22.5)	17.3, 28.8
		Independent (on and off, dressing, wiping)	109 (53.4)	46.6, 60.2
		Missing	0	
	Visit 3	Dependent	24 (13.3)	9.1, 19.1
		Needs some help, but can do something alone	44 (24.4)	18.7, 31.2
		Independent (on and off, dressing, wiping)	112 (62.2)	54.9, 69.0
		Missing	24	
Transfers	Baseline (visit 1)	Unable, no sitting balance	20 (9.8)	6.4, 14.7
		Major help (one or two people, physical), can sit	41 (20.1)	15.1, 26.2
		Minor help (verbal or physical)	30 (14.7)	10.5, 20.3
		Independent	113 (55.4)	48.5, 62.1
		Missing	0	
	Visit 3	Unable, no sitting balance	13 (7.2)	4.2, 12.1
		Major help (one or two people, physical), can sit	34 (18.9)	13.8, 25.3
		Minor help (verbal or physical)	26 (14.4)	10.0, 20.4
		Independent	107 (59.4)	52.1, 66.4
		Missing	24	
Mobility on level surface	Baseline (visit 1)	Immobile or < 50 yards	67 (32.8)	26.8, 39.6
		Wheelchair independent, including corners, > 50 yards	13 (6.4)	3.7, 10.7
		Walks with help of one person (verbal or physical) > 50 yards	49 (24.0)	18.7, 30.3
		Independent (but may use any aid; for example, stick) > 50 yards	75 (36.8)	30.4, 43.6
		Missing	0	
	Visit 3	Immobile or < 50 yards	45 (25.0)	19.2, 31.8
		Wheelchair independent, including corners, > 50 yards	7 (3.9)	1.7, 8.0
		Walks with help of one person (verbal or physical) > 50 yards	44 (24.4)	18.7, 31.2

		Independent (but may use any aid; for example, stick) > 50 yards	84 (46.7)	39.5, 53.9
		Missing	24	
Stairs	Baseline (visit 1)	Unable	70 (34.3)	28.1, 41.1
		Needs help (verbal, physical, carrying aid)	74 (36.3)	30.0, 43.1
		Independent	60 (29.4)	23.6, 36.0
		Missing	0	
	Visit 2	Unable	53 (29.4)	23.3, 36.5
		Needs help (verbal, physical, carrying aid)	57 (31.7)	25.3, 38.8
		Independent	70 (38.9)	32.1, 46.2
		Missing	24	

Percentages are based on the number of non-missing observations in the FAS. FAS, full analysis set.

Table S4. Summary of serious adverse events and causality in patients receiving abobotulinumtoxinA.

**Serious Adverse events Reported Term**	**Events, *n***
**Possibly related, per sponsor assessment**	
Decompensated chronic obstructive pulmonary disease	1
Diarrhea	1
Fall	2
Hypotonia	2
Lost mobility of lower limbs	1
Muscular weakness	2
Pneumonia	1
**Non-related, per sponsor assessment**	
Anemia	1
Bone skull infection	1
Cardiac arrest	1
Cardiac failure	1
Cardiorespiratory arrest	2
Cerebrovascular accident	2
Cognitive worsens	1
Cranial vault infection	1
Dehydration	1
Delirium	1
Dental operation	1
Fall	2
Falling from height	1
Femur fracture	3
Hypertensive crisis	1
Intravesical clot	1
Metabolic acidosis	1
Myocardial infarction	1
Meningitis	1
Passed away *	1
Pneumonia	4
Renal impairment	1
Seizure	1
Sepsis	2
Septic shock	1
Urinary tract infection	2

* Cause of death was unexplained and not reported.

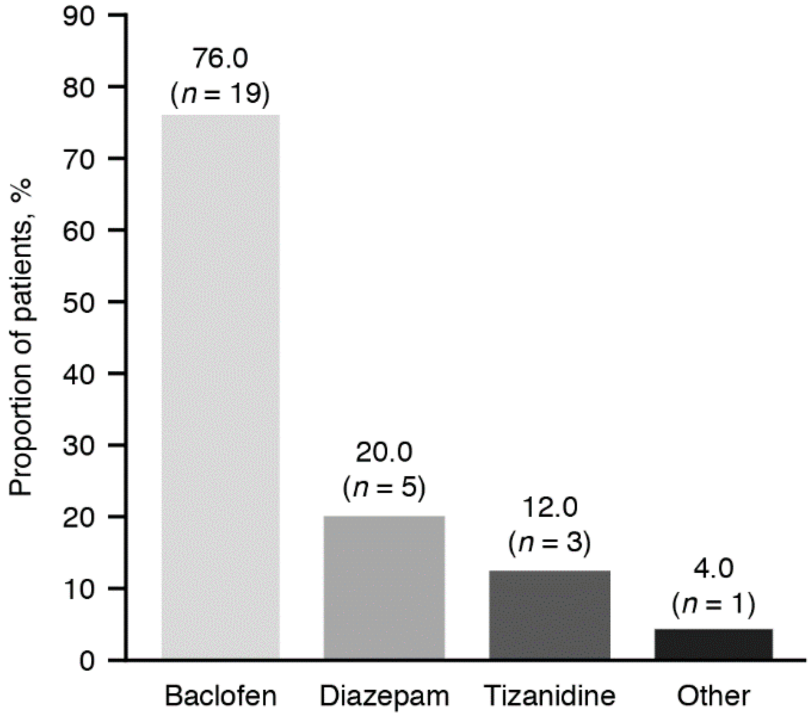

(A)

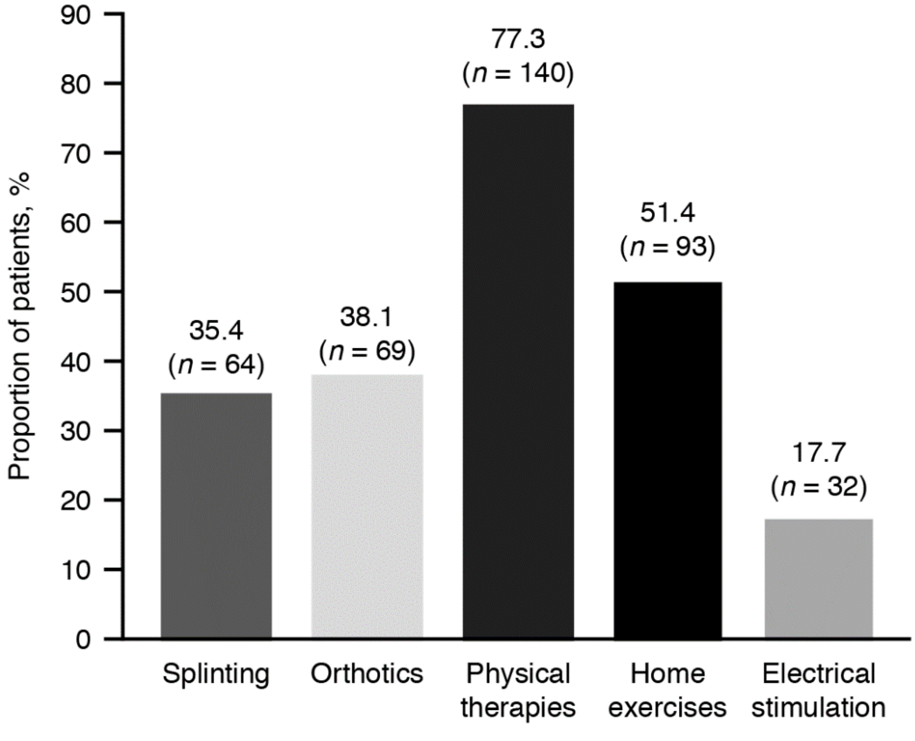

(B)

Figure S1. Previous and concomitant therapies: (**A**) concomitant medications for spasticity (*n* = 25); (**B**) concomitant non-drug therapies for spasticity (*n* = 181) (23 patients in the FAS were missing data, and therefore were not included in this analysis). FAS, full analysis set.

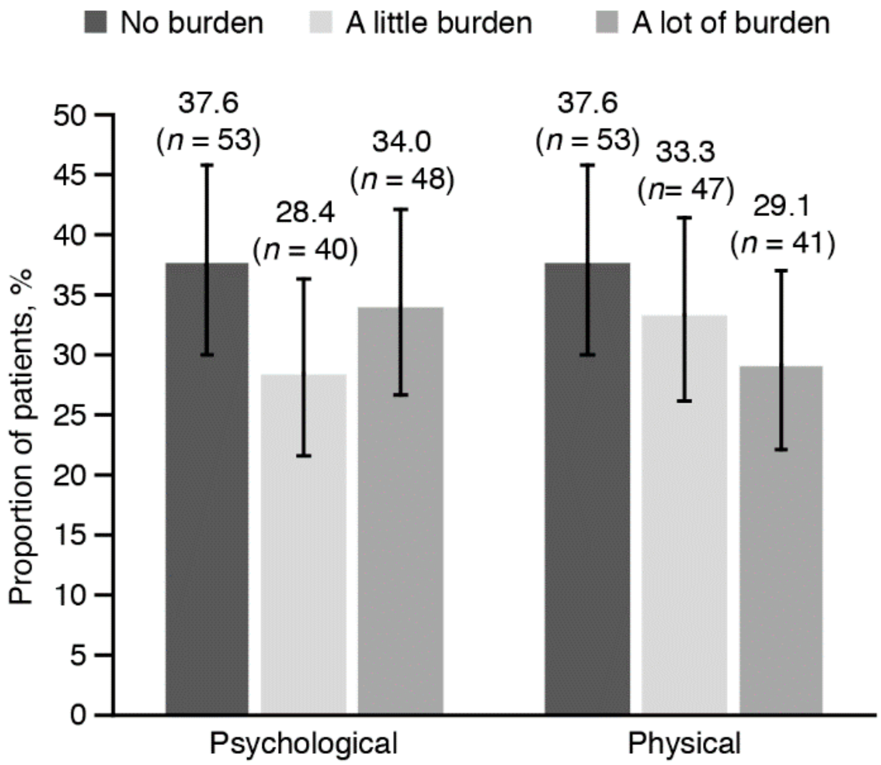

Figure S2. Caregiver's physical and psychological burden at baseline (FAS). Error bars represent 95% CI. Percentages are based on the number of non-missing observations in the FAS; data were available for 141 caregivers. FAS, full analysis set.
